# Adipose triglyceride lipase (Atgl) mediates the antibiotic jinggangmycin-stimulated reproduction in the brown planthopper, *Nilaparvata lugens* Stål

**DOI:** 10.1038/srep18984

**Published:** 2016-01-07

**Authors:** Yi-Ping Jiang, Lei Li, Zong-Yu Liu, Lin-Lin You, You Wu, Bing Xu, Lin-Quan Ge, Qi-Sheng Song, Jin-Cai Wu

**Affiliations:** 1 School of Plant Protection, Yangzhou University, Yangzhou 220059, P.R. China; 2 Division of Plant Sciences, University of Missouri, Columbia, MO 65211, USA

## Abstract

The antibiotic jinggangmycin (JGM) is an agrochemical product widely used in China for controlling rice sheath blight, *Rhizoctonia solani*. Unexpectedly, it stimulates reproduction in the brown planthopper (BPH), *Nilaparvata lugens* (Stål). However, the underlying molecular mechanisms of the stimulation are unclear. The present investigation demonstrates that adipose triglyceride lipase (Atgl) is one of the enzymes involved in the JGM-stimulated reproduction in BPH. Silence of *Atgl* in JGM-treated (JGM + dsAtgl) females eliminated JGM-stimulated fecundity of BPH females. In addition, *Atgl* knockdown significantly reduced the protein and glycerin contents in the ovaries and fat bodies of JGM + dsAtgl females required for reproduction. We conclude that Atgl is one of the key enzymes responsible for JGM-stimulated reproduction in BPH.

Antibiotic products in agrochemicals are generally considered as environmentally friendly pest control technologies. The antibiotic jinggangmycin (JGM), a product of *Streptomyces* var. *jinggangensis* developed in China^1^, is widely used in China for controlling rice sheath blight, *Rhizoctonia solani,* with foliar spray at the commercial rate of 125–175 g.a.i ha^−1^. However, it unexpectedly exerts substantial negative effects on agro-ecosystems. For example, JGM stimulated the reproduction and increased vitelline content and vitellogenin gene expression of the brown planthopper (BPH), *Nilaparvata lugens* (Stål) (Homoptera: Delphacidae)^2^, but reduced the content of the resistance-related substance oxalic acid in rice plants and the export rates of photosynthetic products in rice leaves^3^,^4^. As a result, JGM increased the susceptibility of rice to BPH^5^. However, there is little information on the molecular mechanisms or the ecological consequences of JGM-induced changes in BPH reproduction. Understanding of the underlying mechanisms or the consequences of this effect would be highly valuable for pest management in agro-ecosystems because JGM has been widely applied in China to control rice sheath blight, *R. solani,* two or three times each growing season at commercial (or greater) rates for more than two decades. It has been overused because of its very low toxicity to mammals (LD_50_ > 20,000 mg/kg for rats)^6^.

BPH is a serious rice pest in Asia and China^7^,^8^,^9^. Some, but not all, pesticides stimulate BPH reproduction^2^,^10^,^11^ and result in a resurgence of its occurrence^12^,^13^. Previous studies on pest resurgence induced by pesticides focused mainly on insecticides. In fact, some of herbicides (e.g., butachlor, metolachlor and bentazone) and fungicide jinggangmycin, could also stimulate the reproduction of BPH^2^,^5^,^14^. In addition to stimulate female reproduction, some insecticides (e.g., triazophos and deltamethrin) could also stimulate the reproduction of BPH males^15^,^16^,^17^. Thus, BPH is a classic example of a pesticide-induced resurgent pest.

The present study investigated differentially expressed genes in JGM-treated adult BPH females based on the gene expression profiles, and identified adipose triglyceride lipase gene (*Atgl*) as the target for gene silencing by RNAi because it was highly expressed in JGM-treated females and because it plays critical roles in energy balance leading to reproduction. Atgl acts as a hydrolase in lipolysis and decomposes triglycerides into diglyceride and fatty acids, working together with a series of enzymes^18^,^19^,^20^. Fatty acids are metabolized into energy via β-oxidation^21^. Many investigations have demonstrated that oocyte maturation is related to metabolic energy^22^. Therefore, Atgl plays an important role in the maintenance of energy balance by regulating triglyceride decomposition and is an ideal RNAi target for investigating its role in JGM-stimulated BPH reproduction. The objectives of the present study were to examine the molecular evidence that JGM stimulated reproduction in BPH and to provide new insights into the resurgence of this pest.

## Results

### Selection of differentially expressed genes from JGM-induced gene expression profiles

**Figure 1:**
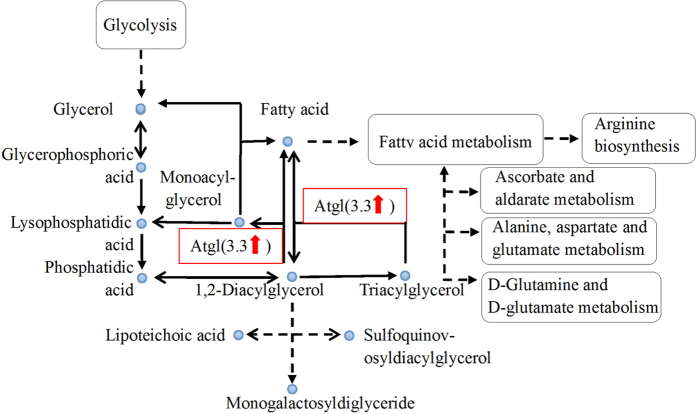
Simple pathway about glycerol metabolism. Atgl is adipose triglyceride lipase; arrow represents up-expression fold for JGM-treated group.

We compared gene expression levels between the JGM-treated and control females 2 days after adult emergence and identified 144 differentially expressed genes, of which 84 were significantly up-regulated and 60 down-regulated. Based on these results, we targeted *Atgl* for RNAi analysis of the JGM-induced molecular influence on the reproduction and biochemistry of BPH because *Atgl* was upregulated by 3.3 folds in the JGM-treated females compared to that in the control females and because it plays an important role in the energy metabolism pathway required for reproduction (Fig. 1).

### Influence of Atgl silencing on the reproduction of females

**Figure 2:**
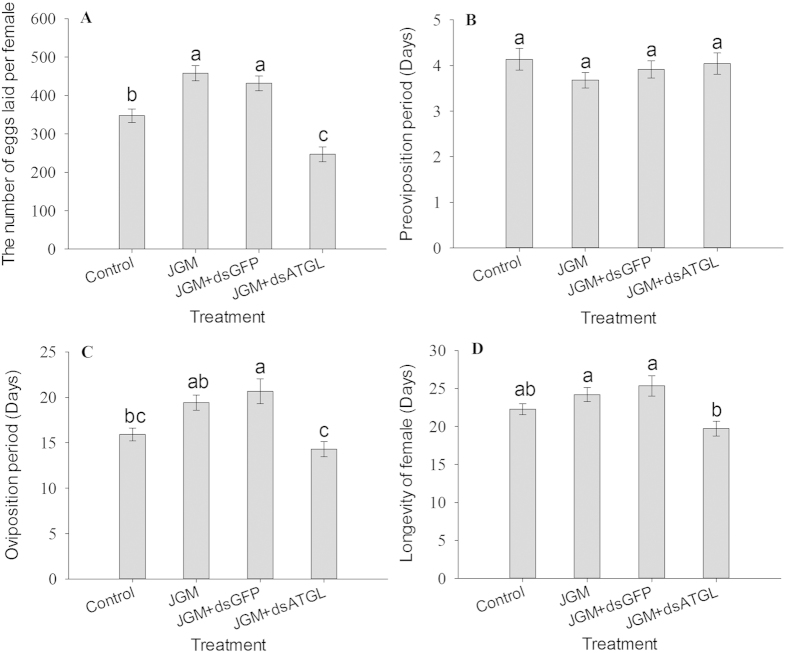
Dietary dsAtgl influenced BPH reproductive parameters. (**Panel** **A**) The number of eggs laid per female; (**Panel** **B**) Pre-oviposition period (days); (**Panel** **C**) Oviposition period (days); (**Panel** **D**) Adult female longevity (days). The histogram bars represent the mean ± SEM of >3 biological replicates. The bars annotated with the same letter are not significantly different.

*Atgl* silencing significantly influenced the number of eggs laid by the JGM-treated (JGM + dsAtgl) females, the oviposition period and the longevity (Fig. 2) (Table 1), but did not influence the pre-oviposition period. Multiple comparisons of the means showed that the number of eggs laid by the JGM + dsAtgl females was significantly lower when compared with that of the JGM females (without *Atgl* silencing), the JGM + dsGFP negative control, and the control without JGM treatment, decreasing by 46.1% (from 458.36 to 246.84), 42.9% (from 431.9 to 246.84) and 28.9% (from 347.3 to 246.8), respectively, indicating that *Atgl* silencing eliminated JGM-stimulated reproduction. The oviposition period of the JGM + dsAtgl females was significantly shorter than that of the JGM females (without *Atgl* silencing) and the JGM + dsGFP control, decreasing by 26.4% and 30.9%, respectively. The longevity of the JGM + dsAtgl females was significantly lower than that of JGM females (without *Atgl* silencing) and the JGM + dsGFP control, with a decrease of 18.5% and 22.2%, respectively.

### Influence of Atgl silencing on protein content in the ovaries and fat bodies of JGM-treated females

**Figure 3:**
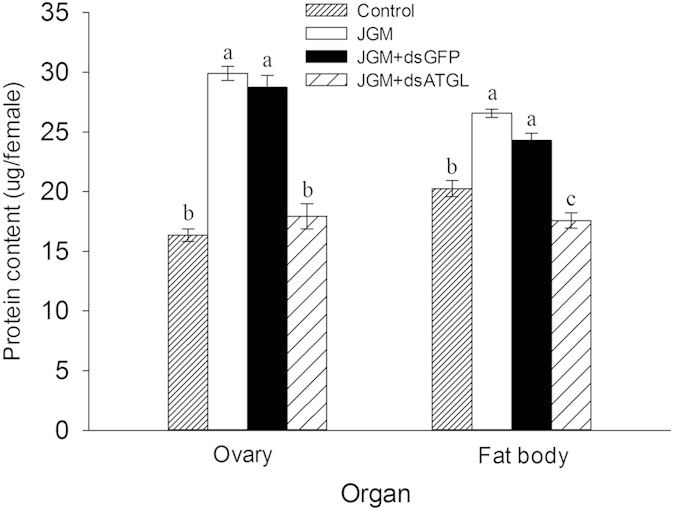
Dietary dsAtgl influenced protein content in the ovaries and fat bodies of BPH. The histogram bars represent the mean (n = 3 independent biological experiments) ± SEM. The bars annotated with the same letter within the same organ are not significantly different.

*Atgl* silencing significantly influenced the protein content in the ovaries and fat bodies of the JGM + dsAtgl females (Fig. 3) (Table 1). Multiple comparisons of the means showed that the protein content in the ovaries of the JGM + dsAtgl females was significantly lower than that of the JGM females (without *Atgl* silencing) and that of the JGM + dsGFP control, with a decrease of 40.0% and 37.5%, respectively. The protein content in the fat bodies of the JGM + dsAtgl females was significantly lower than that of the JGM females (without *Atgl* silencing), the JGM + dsGFP control, and the untreated control, with a decrease of 33.8%, 27.7%, and 13.2%, respectively.

### Influence of Atgl silencing on the soluble sugar content in the ovaries and fat bodies of JGM-treated females

**Figure 4:**
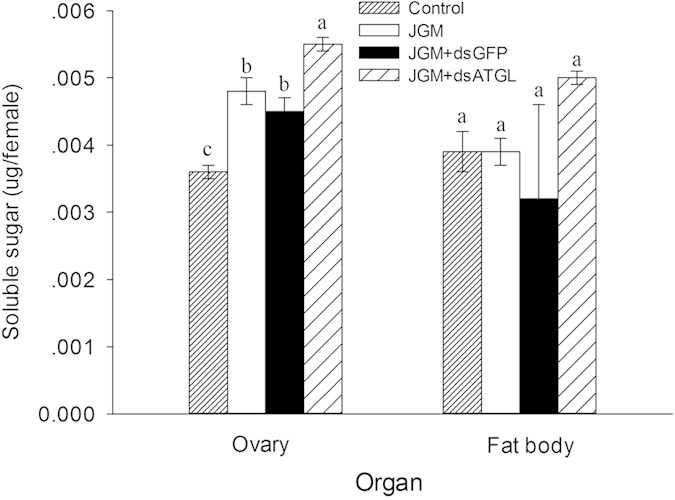
Dietary dsAtgl influenced soluble sugar in the ovaries and fat bodies of BPH. The histogram bars represent the mean (n = 3 independent biological experiments) ± SEM. The bars annotated with the same letter within the same organ are not significantly different.

*Atgl* silencing significantly influenced the soluble sugar content in the ovaries, but not in the fat bodies of the JGM + dsAtgl females (Fig. 4) (Table 1). Multiple comparisons of the means showed that the soluble sugar content in the ovaries of the JGM + dsAtgl females was significantly higher when compared to than that of the JGM females (without *Atgl* silencing), the JGM + dsAtgl control, and the untreated control, with an increase of 14.6%, 22.2%, and 52.8%, respectively.

### Influence of Atgl silencing on the glycerin content of the JGM-treated females

**Figure 5:**
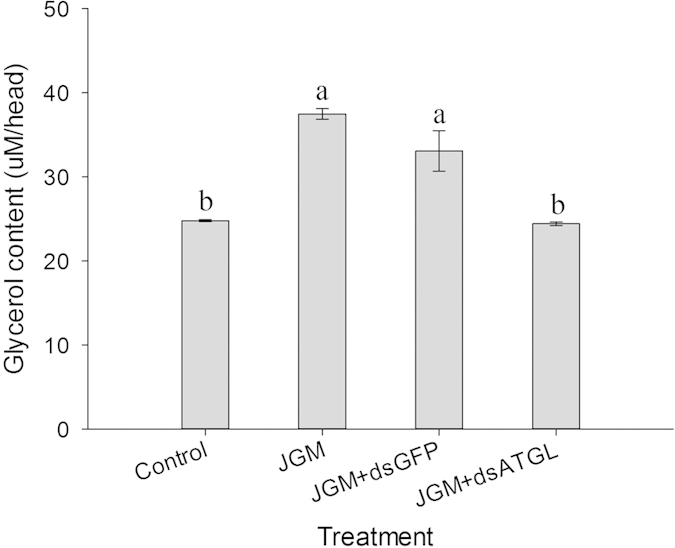
Dietary dsAtgl influenced the glycerin content of JGM-treated BPH females. The histogram bars represent the values indicated on the Y-axes, and the error bars represent SEM. The bars annotated with the same letter within the same organ are not significantly different.

*Atgl* silencing significantly influenced the glycerin content in the JGM + dsAtgl females (Fig. 5) (Table 1). Multiple comparisons of the means showed that the glycerin content in the JGM + dsAtgl females was significantly lower than that of the JGM females (without *Atgl* silencing) and the JGM + dsGFP control, with a decrease of 34.8% and 26.1%, respectively.

### Influence of Atgl silencing on the body weight of the JGM-treated females

**Figure 6:**
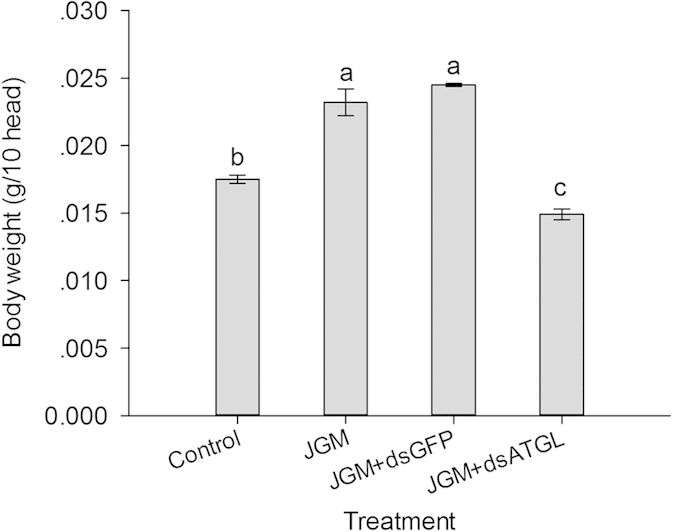
Dietary dsAtgl influenced body weight of BPH. The histogram bars represent the mean ± SEM of three independent replicates, each with 10 females. The bars annotated with the same letter are not significantly different.

*Atgl* silencing significantly influenced the body weight of the JGM + dsAtgl females (Fig. 6) (Table 1). Multiple comparisons of the means showed that the body weight of the JGM + dsAtgl females was significantly lower when compared with that of the JGM females (without *Atgl* silencing), the JGM + dsGFP control, and the untreated control, with a decrease of 35.8%, 39.2%, and 14.9%, respectively.

### qPCR analysis of transcription profiles

**Figure 7:**
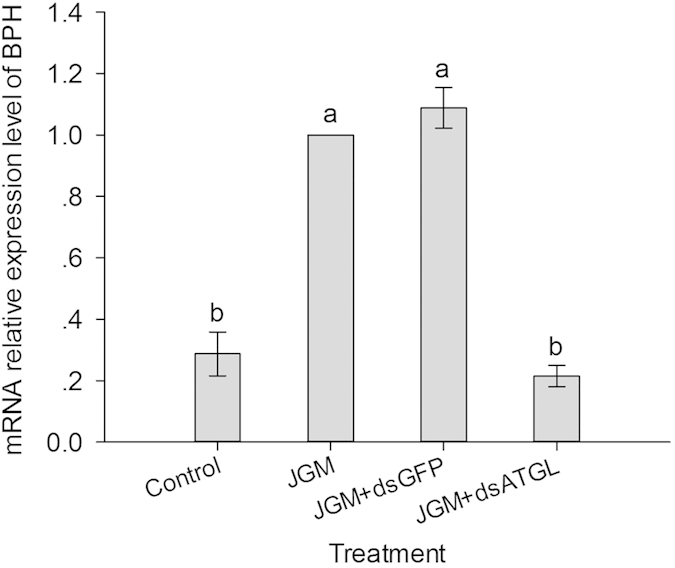
qPCR analysis of the mRNA expression levels of Atgl. The histogram bars represent the mean ± SEM of three independent biological replicates. The bars annotated with the same letter are not significantly different.

The dietary dsRNA treatment led to the down-regulation of *Atgl* expression in BPH (Fig. 7) (Table 1). The *Atgl* expression level of the JGM + dsAtgl group was significantly lower than that of the JGM and JGM + dsGFP groups, with a decrease of 78.5% and 80.3%, respectively.

## Discussion

We have previously reported the JGM-stimulated reproduction in BPH^12^,^23^, but the underlying molecular mechanisms were not clear. The present investigation revealed several points relevant to the underlying mechanisms. First, JGM treatment induced a set of differentially expressed genes, including the up-regulation of *Atgl*; second, the *Atgl*-silencing reduced the number of eggs laid by JGM treated females, eliminating JGM-stimulated reproduction; third, *Atgl*-silencing significantly reduced the protein content required for ovarian development, but increased soluble sugar content in the ovaries and fat bodies of the JGM treated females. These findings showed that Atgl affects the JGM-stimulated reproduction in BPH. Undoubtedly, *Atgl* is not the only gene involved in mediating the effects of JGM on the reproduction of BPH, however, we believe that *Atgl* is an important mediator because it is related to energy metabolism. Physiologically and biochemically, it has been demonstrated that insecticide treatments increased the soluble sugar and fat body contents of BPH and resulted in an increase in reproduction^10^. The antibiotic JGM treatment could also significantly increase the protein content in the ovaries and fat bodies of BPH^24^. These exogenous factors may influence the feeding and survival of BPH on rice plants, thus affecting metabolic capacity^3^,^5^,^14^. The up-regulation of *Atgl* enhanced the metabolic capacity and resulted in the increase of BPH reproductive capacity. Therefore, *Atgl*-silencing decreased the protein content in the ovaries and fat bodies of BPH required for ovarian development, resulting in decreased fecundity of BPH.

For insects, fat body is an important tissue for insect growth, development, reproduction and other metabolic activities. Triglycerides and glycogen are the main energy storage materials in fat body^25^, providing material and energy for insect growth, development and reproduction. As the first enzyme in the lipolysis process, Atgl is a speed-limiting enzyme and plays an extremely important role in lipolysis. The present findings showed that the protein content in the ovaries and fat bodies of the JGM + dsAtgl females decreased, but the soluble sugar content increased, the later may be associated with the decline of lipid decomposition rate due to *Atgl* silencing. The declines in proteins and fatty acids of the *Atgl*-silenced females resulted in a decrease in the number of eggs laid because vitellogenin synthesis requires the building bricks, amino acids from protein metabolism, and metabolic energy from fat acid metabolism. Furthermore, vitellogenin of insects is mainly synthesized in fat bodies, transferred to ovaries and absorbed by oocytes, and these processes demand high energy. We also measured their body weight and discovered that body weight of the JGM + dsAtgl females was significantly lower than that of JGM and JGM + dsGFP control. Although body weight is not directly related to the fecundity, however, we speculate that the decline in the number of eggs laid, protein content, glycerin content of the JGM + dsAtgl females may be associated with body weight, resulting from the impaired food uptake.

Atgl is expressed in many organs or tissues, including fat, myocardial and testis tissues in higher animals, of which fat tissue has the highest expression level^26^. However, there have been little studies of Atgl in insects. We tested whether Atgl is important in the triglyceride and fatty acid pathway through analysis of the gene expression profiles of BPH (Fig. 1) because fatty acids have been demonstrated to be closely associated with the reproduction of *Drosophila*^27^,^28^,^29^. Our results from BPH confirm the role of Atgl-mediated triglyceride and fatty acid metabolism in insect reproduction. The functions of other differentially expressed genes related to JGM-stimulated reproduction in BPH require further investigation. Since JGM is an important agricultural product associated with sustaining long-term food and ecology securities, evaluation of its impact on agroecosystem will provide guideline for safe use of JGM in pest management program.

## Materials and Methods

### Rice variety, insects, and pesticides

The rice (*Oryza sativa* L.) variety Ningjing 2 (japonica rice) was used in each trial. This variety of rice was selected because it is commonly planted in Jiangsu province, China. The seeds were sown outdoors in standard rice-growing soil in cement tanks (length 200 cm, width 100 cm, and height 60 cm). When the seedlings reached the 6-leaf stage, they were transplanted into plastic pots (diameter 16 cm, height 30 cm), with 4 hills per pot and 3 plants per hill. The rice plants used in the experiments were at the tillering stage.

BPHs used in the experiments were obtained from a stock population maintained at the China National Rice Research Institute (CNRRI; Hangzhou, China). BPH were reared at Yangzhou University in an insect nursery consisting of rice plants covered with cages under natural conditions in cement tanks from April to October and were placed in a greenhouse during winter. Prior to the experiments, the BPH colony was allowed to reproduce for two generations in an insectary with a controlled temperature and photoperiod (28 ± 2 °C and L 14 : D10) without insecticide application to ensure that the population was large enough before the insects were released. Technical grade JGM (C_20_H_35_O_13_N) (61.7% a.i.) (Qianjiang Biochemistry Co. Ltd., Zhejiang, China) was used in all trials.

### Experiments

Three hundred third-instar nymphs per caged pot were released onto the potted plants at the tillering stage. The cages were opened 24 h after the insects were released, and the potted plants were sprayed with 200 ppm JGM after adding 10% emulsifier (Tween 20, an adjuvant) (Jinglin Chemical Co. Ltd., Nanjing, Jiang, China) to reduce the surface tension on the leaf using a Jacto sprayer (Maquinas Agricolas Jacto S.A., Brazil) equipped with a cone nozzle (1-mm diameter orifice) at a pressure of 45 psi, which yielded a flow rate of 300 ml/min. The control plants at the same developmental stage as the treated plants were sprayed with tap water and non-active substances (emulsifier). Each treatment was replicated three times (3 pots), and the pots were distributed in a randomized pattern. The final fifth-instar nymphs (72 h after spray) on the treated and control plants were collected for the RNAi experiment. In addition, the nymphs were collected and placed into glass jars (10 cm diameter, 12 cm height) with untreated rice plants to complete their development in an artificial climate box (Model: RXZ 328A) (Jiangnan Instrument Factory, Ningbo, Zhejiang, China) at 26 ± 2 °C and L14 : D10. The development of the nymphs was recorded until adult emergence. At 2 days after emergence (2 DAE), the adult females were collected to analyze their gene expression profiles. Many previous investigations demonstrated that third instar nymphs are the key stage to pesticide-stimulated reproduction in BPH^10^,^11^,^30^.

### Analysis of JGM-induced gene expression profiles

Fifty virgin BPH females (2 DAE) developed from the third instar nymphs that had been treated with JGM or control were placed in a −80 °C freezer for RNA extraction, and analyzed for differentially expressed genes using a differential gene expression approach. The total RNA was isolated from BPH using the SV Total RNA Isolation System (Promega). The samples were treated with DNase, and the RNA quantity was evaluated using a spectrophotometer (NanoDrop 2000, Thermo). RNA quality was evaluated using a 2100 Bioanalyzer (Agilent) according to the Illumina instructions.

mRNA was isolated from 6 μg of total RNA from each sample using oligo (dT) magnetic beads, and then double-stranded cDNA was synthesized after reverse transcription using oligo (dT) primers bound to magnetic beads. Two restriction enzymes were used to generate sequencing tags. The cDNA was digested with *NlaIII*, and then Illumina adapter 1 was annealed to the 5′ ends of the cDNA fragments attached to the magnetic beads. The junction of Illumina adapter I and CATG generates the MmeI recognition site. MmeI was used to cut the cDNA fragments 17 bp downstream of the CATG site. After collecting the cDNA fraction with magnetic beads, Illumina adapter 2 was ligated at the 3′ ends of the tags. After fifteen cycles of linear PCR amplification, 105 bp fragments were purified on 6% TBE PAGE. After denaturation, the single-strand sequences were fixed onto an Illumina HiSeq TM 2000 chip. Each molecule grew into a single-molecule cluster-sequencing template via *in situ* amplification, and four color-coded nucleotides were added to the chip for sequencing by synthesis. The adaptor sequences, low quality tags (tags containing unknown nucleotides and tags with more than 50% bases with a quality value less than 10), empty reads (reads with only 3′ adaptor sequences but no tags), tags that were either too long or too short, and single-copy (probable sequencing error) tags were filtered out to generate clean tags. The sequencing data were evaluated by assessing the distribution of tag expression, the saturation of sequencing data, and experimental reproducibility.

The clean tags were mapped to the BPH reference genes at Beijing Genomics Institute (BGI, Shenzhen, China). A preprocessed reference database of all the possible CATG + 17 nucleotides tag sequences was created using the reference genes. Subsequently, all clean tags were mapped to the reference database using SOAP2^31^, allowing no more than 1 nucleotide mismatch. The clean tags that mapped to multiple genes in the reference sequences were filtered out. The remaining clean tags were unambiguous clean tags. For gene expression analysis, the number of unambiguous clean tags for each gene was calculated and then normalized to the number of transcripts per million (TPM) clean tags^32^.

We recorded the differential gene expression across the samples using an algorithm^21^. To compare the differences in gene expression between the samples, the tag frequency in each library was analyzed according to Mortazavi *et al.*^33^. The false discovery rate (FDR) was used to determine the threshold P value in multiple tests. A FDR ≤ 0.001 and an absolute value of the log2 ratio ≥ 1 were used as the thresholds to determine the significant differences in gene expression. We used the following equation to quantify the relative gene expression:


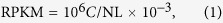


where C = the number of reads that only map to one unigene, N = total number of C, and L = the number of bases in one unigen.

The *P* value was calculated for gene ontology (GO) (http://www.geneontology.org/) with a Bonferroni correction. A corrected *P* value ≤ 0.05 was the threshold for the significant enrichment of the gene sets. WEB Gene Ontology Annotation Plot (WEG) software was used for visualizing, comparing and plotting GO annotation results^34^. Pathway enrichment analysis identified significantly enriched metabolic pathways or signal transduction pathways using the KEGG database^35^. Pathways with a *Q* value ≤ 0.05 were significantly enriched pathways.

### dsRNA synthesis

Based on our transcriptome data, we selected *Atgl* for RNAi silencing because it was up-regulated (3.3-fold) in the JGM-treated BPHs. For dsRNA synthesis, a 248 bp fragment of BPH *Atgl* with the T7 primer sequence at the 5′ ends (Table 1) was amplified. The amplification reactions were programmed for 35 cycles at 95 °C for 1 min, 58 °C for 40 min, and 72 °C for 1 min, with a final extension step of 72 °C for 10 min. The identities of the PCR products were verified by DNA sequencing. Those sequences in which products from the forward and reverse sequencing results aligned well (98–99%) were used for dsRNA synthesis. The green fluorescent protein (GFP)-encoding gene (ACY56286) was used as control dsRNA, and the primers GFP-F and GFP-R were used to amplify the GFP fragment (688 bp) (Table 2). The dsRNAs were prepared using the T_7_ RiboMax Express RNAi System (Promega, Madison, WI, USA). The sense and antisense dsRNAs generated in separate 20 μl total reaction volumes were annealed by mixing both transcription reactions incubated at 70 °C for 10 min, and then cooled down to room temperature over a 20 min time period. A 2 μl dilution of RNase A solution (1 : 200) and a 2 μl RNase-free DNase sample were added to the mixture solutions of each transcription reaction and bathed in 37 °C water for 30 min. The dsRNA was precipitated with 110 μl 95% ethanol and 4.4 μl 3 M sodium acetate (pH 5.2), washed with 0.5 ml 70% ethanol and dried at room temperature before dissolving in 50 μL nuclease-free water. The purified dsRNAs were quantified at 260 nm and examined on agarose gels to ensure their integrity.

### Silencing Atgl

Insects were released onto potted rice and treated with JGM as described above. The experimental and control third-instar nymphs (20/treatment) were collected 3 days after the JGM treatments. They were treated with RNAi according to Dong *et al.* (2011)^36^. The dsRNA at the final concentration of 0.125 μg/μl was added to the artificial diet (40 μl). The diet solution was changed every 2 days. After four days of feeding on the dsRNA-laced medium, nymphs reached their fifth instar and were transferred individually into a glass jar containing untreated rice stems. The newly emerged females from these nymphs were transferred to culture boxes. Two hundred adult females were collected separately at 2 DAE, and protein content, soluble sugar content, body weight, glycerin content, and *Atgl* expression level were determined. A pair (♀×♂) from the same replicate was placed in a glass cup containing untreated rice stems for egg laying. The rice stems were replaced every 2 days. The eggs laid on each rice stem were counted under a microscope. Twenty copulating pairs were maintained to record duration of the pre-oviposition period, oviposition period, adult female longevity, and fecundity for each copulating pair.

### Quantitative real-time PCR (qPCR) analysis

Total RNA from the RNAi and control BPHs was extracted and reverse transcribed. Portions (2 μl) of the synthesized first-strand cDNA were amplified by qPCR in 20 μl reaction mixtures using a CFX 96 PCR system (Bio-Rad, California, USA) with this program: 94 °C for 2 min, followed by 40 cycles of 94 °C for 5 s, 49.4 °C for 30 s, and 72 °C for 30 s. The BPH actin-1 gene was used as a reference gene. The primers used for qPCR analysis are listed in Table 1. After amplification, a melting curve analysis was performed in triplicate, and the results were averaged. The values were calculated using three independent biological samples, and the 2^−ΔΔCT^ method of Livak and Schmittgen^37^ was used for analyzing the relative expression levels of *Atgl*.

### The influence of Atgl silencing on the biochemistry of BPH

The procedure described in Gong *et al.*^38^ was followed to measure the protein contents in the fat bodies and ovaries of BPH with Coomassie Brilliant Blue G 250 (Shanghai Chemical Agent Co. Ltd., Shanghai, China). The fat bodies and ovaries were dissected under a zoom-stereomicroscope (model XTL20, Beijing Tech Instrument Co. Ltd., Beijing, China) in a cooled petri dish. The ovaries and fat bodies of adult females were removed and placed in separate, pre-weighed, ice-cold centrifuge tubes, re-weighed using a Mettler-Toledo electronic balance (EC 100 model; 1/10000 g sensitivity), homogenized on ice after adding 5 ml phosphate buffered saline (PBS), and centrifuged at 10,000 rpm at 4 °C for 20 min. The supernatant was collected after filtering the upper fat layer with glass fibers. A standard curve was established based on a standard protein (bovine serum albumin, from Shanghai Biochemistry Research Institute, Shanghai, China). The absorbance at 595 nm was determined in a UV755 B spectrometer (Shanghai Precision Instrument Co. Ltd., Shanghai, China). The protein content in the sample solution was calculated according to a standard curve.

The soluble sugar level was measured using the anthrone method^39^. The adult females were placed in a mortar and ground with 10 ml of 99.7% alcohol. The sample solution was put into a test tube, incubated for 30 min at 80 °C, cooled down, and centrifuged at 2,000 rpm for 20 min. The volume of supernatant was measured. The extraction process was repeated three times. The volume of supernatant was adjusted to 50 ml with distilled water. One milliliter of supernatant was placed into a 10 ml test tube, to which 1 ml of distilled water and 4 ml of anthrone were added. The test tube was placed in a water bath, boiled for 15 min, and cooled down. The absorbance at 620 nm was detected with the 722 spectrometer (Third Analytical Instrument Co. Ltd., Shanghai, China). Glucose was used to construct a standard curve.

The glycerin content in adult females at 2 DAE was measured using a Glycerol Assay kit (Nanjing Jiangcheng Bioengineering Institute, Nanjing, Jiangsu, China). Fifteen females were used for each replicate. Each treatment was repeated three times.

For body weight measurement, ten females at 2 DAE were used as a replicate for each treatment and control group. Adult females were placed in pre-weighed centrifuge tubes and then weighed using a Mettler-Toledo electronic balance (EC100 model; 1/10,000 g sensitivity). Each treatment and control group was replicated three times.

### Statistical analysis

The normal distributions and homogeneity of variances (determined using the Bartlett test) were verified before performing analysis of variance (ANOVA) tests. A one-way ANOVA was performed for the number of eggs laid, the pre-oviposition period, the oviposition period, the longevity of the females, the contents of protein, soluble sugar and glycerin, and the body weight. Multiple comparisons of the means were conducted based on Fisher’s protected least significant difference (PLSD) test. All analyses were conducted using the data processing system (DPS) of Tang *et al.* (2002)^40^.

## Additional Information

**How to cite this article**: Jiang, Y.-P. *et al.* Adipose triglyceride lipase (Atgl) mediates the antibiotic jinggangmycin-stimulated reproduction in the brown planthopper, *Nilaparvata lugens* Stål. *Sci. Rep.* **6**, 18984; doi: 10.1038/srep18984 (2016).

## Figures and Tables

**Table 1:** *F*-statistics for all experiments.

Experiment	*F*-Statistic
The number of eggs laid by JGM-treated *Atgl-silenced* BPH females	*F* = 25.75, df = 3, 92, *P* = 0.0001
Pre-oviposition period of JGM-treated *Atgl-silenced* BPH females	*F* = 0.887, df = 3, 92, *P* = 0.4509
Oviposition period of JGM-treated *Atgl-silenced* BPH females	*F* = 9.314, df = 3, 92, *P* = 0.0001
Longevity *of Atgl-silenced* BPH females	*F* = 5.86, df = 3, 92, *P* = 0.001
Protein content in the ovaries of *Atgl-silenced* BPH females	*F* = 72.7, df = 3, 8, *P* = 0.0001
Protein content in the fat bodies of *Atgl-silenced* BPH females	*F* = 48.6, df = 3, 8, *P* = 0.0001
Soluble sugar content in the ovaries of *Atgl-silenced* BPH females	*F* = 31.3, df = 3, 8, *P* = 0.0001
Glycerin content in the ovaries of *Atgl-silenced* BPH females	*F* = 25.9, df = 3, 8, *P* = 0.0002
Body weight of *Atgl-silenced* BPH females	*F* = 69.9, df = 3, 8, *P* = 0.0001
mRNA expression levels of *Atgl* (qPCR)	*F* = 79.7, df = 2, 8, *P* = 0.0001

**Table 2:** PCR primers used in this study.

Primer	Primer sequence
For quantitative real-time PCR	
Atgl*-*F	5′-GCGACCATTTATCCCATTA-3′
Atgl*-*R	5′-GACTTCACCAGCCCAGAC-3′ 247 bp
Actin-F	5′-TGGACTTCGAGCAGGAAATGG-3
Actin-R	5′-ACGTCGCACTTCAGATCGAG-3′ 200 bp
For Atgl dsRNA synthesis	
Atgl-F	5′- GCGACCATTTATCCCATTA-3′
Atgl-R	5′-TGACTTCACCAGCCCAGAC-3′ 248 bp
For GFP dsRNA synthesis	
GFP-F	5′- AAGGGCGAGGAGCTGTTCACCG-3′
GFP-R	5′-CAGCAGGACCATGTGATCGCGC-3 688 bp
